# Fostering Responsible Innovation in Health: An EvidenceInformed Assessment Tool for Innovation Stakeholders

**DOI:** 10.34172/ijhpm.2020.34

**Published:** 2020-03-15

**Authors:** Hudson P. Silva, Andrée-Anne Lefebvre, Robson R. Oliveira, Pascale Lehoux

**Affiliations:** ^1^Public Health Research Institute, University of Montreal, Montreal, QC, Canada.; ^2^School of Public Health, University of Montreal, Montreal, QC, Canada.

**Keywords:** Responsible Innovation, Health Technology, Assessment Tool, Multi-criteria Decision Analysis, Sustainability, Equity

## Abstract

**Background:** Responsible innovation in health (RIH) emphasizes the importance of developing technologies that are responsive to system-level challenges and support equitable and sustainable healthcare. To help decision-makers identify whether an innovation fulfills RIH requirements, we developed and validated an evidence-informed assessment tool comprised of 4 inclusion and exclusion criteria, 9 assessment attributes and a scoring system.

**Methods:** We conducted an inter-rater reliability assessment to establish the extent to which 2 raters agree when applying the RIH Tool to a diversified sample of health innovations (n=25). Following the Tool’s 3-step process, sources of information were collected and cross-checked to ensure their clarity and relevance. Ratings were reported independently in a spreadsheet to generate the study’s database. To measure inter-rater reliability, we used: a non-adjusted index (percent agreement), a chance-adjusted index (Gwet’s AC) and the Pearson’s correlation coefficient. Results of the Tool’s application to the whole sample of innovations are summarized through descriptive statistics.

**Results:** Our findings show complete agreement for the screening criteria, "almost perfect" agreement for 7 assessment attributes, "substantial" agreement for 2 attributes and "almost perfect" agreement for the RIH overall score. A large portion of the sample obtained high scores for 6 attributes (health relevance, health inequalities, responsiveness, level and intensity of care and frugality) and low scores for 3 attributes (ethical, legal, and social issues [ELSIs], inclusiveness and eco-responsibility). At the rating step, 88% of the innovations had a sufficient number of attributes documented (≥ 7/9), but the assessment was based on sources of moderate to high quality (mean score ≥ 2 points) for 36% of the sample. While "Almost all RIH features" were present for 24% of the innovations (RIH mean score between 4.1-5.0 points), "Many RIH features" were present for 52% of the sample (3.1-4.0 points) and "Few RIH features" were present for 24% of the innovations (2.1-3.0 points).

**Conclusion:** By confirming key aspects of the RIH Tool’s reliability and applicability, our study brings its development to completion. It can be jointly put into action by innovation stakeholders who want to foster innovations with greater social, economic and environmental value.

## Introduction

Successful and sustainable health systems are characterized by healthy individuals, superior care, fairness of treatment, affordability, acceptability for patients and health professionals, and adaptability to epidemiologic, demographic, scientific, and technological changes.^1^ Although new technologies can support such characteristics, current ways of designing and commercializing health innovations combine for-profit business models with the financialized logic of venture capital, which tend to generate innovations with a marginal added value that health systems can hardly afford.^2^ The implementation of complex and labour-intensive technologies also raises major system-level challenges in terms of service delivery, human resources, governance and the maintenance of infrastructure and equipment.^3,4^ In this constantly evolving context, decision-makers face a formidable challenge, which is to improve the basis upon which they decide whether to fund, cover or reimburse new health technologies while remaining able to address other pressing population health and social care challenges.

The use of multi-criteria decision analysis (MCDA) to support decision-making in health has increased over the last decade.^5-10^ Several public and private healthcare organizations and governmental agencies have used and proposed MCDA as an approach to assess new health technologies, prioritize investments in public health interventions, assess orphan drugs and develop universal health coverage benefit packages.^9^ One well known MCDA model is the EVIDEM framework, which aims to foster “sustainable health and well-being for all” by combining 20 ethical-normative and contextual-feasibility criteria to set priorities among “all types of healthcare interventions, for all levels of decision, and across the globe.”^11^ However, applications of MCDA in healthcare usually rely on “downstream” criteria, which are best documented once the innovations have hit the market and are more widely used,^9,12^ and pay little attention to the “upstream” characteristics that condition the purposes, functions and costs of innovations before they make their way into health systems.

Responsible research and innovation (RRI), which emerged under the impetus of innovation scholars and policy-makers, refocuses our attention upstream in order to foster the development of innovations that are ethically acceptable, sustainable and socially desirable.^13^ More specifically, 4 forward-looking processes are emphasized: anticipation of risks, impacts and unintended consequences; reflexivity regarding value systems and social practices governing innovation; inclusiveness in innovation development processes; and responsiveness to emerging knowledge, outcomes and shifting contexts.^14^ RRI also deliberately seeks to align innovation with economic, social or environmental challenges such as the Sustainable Development Goals of the United Nations.^15^

Recent efforts were made to adapt the RRI principles to the specificities of the healthcare sector and develop a responsible innovation in health (RIH)framework. For Silva et al,^16^ “RIH consists of a collaborative endeavour wherein stakeholders are committed to clarify and meet a set of ethical, economic, social and environmental principles, values and requirements when they design, finance, produce, distribute, use and discard sociotechnical solutions to address the needs and challenges of health systems in a sustainable way.” Structured around this definition, a RIH framework was developed and is comprised of 5 value domains and 9 attributes that are considered throughout the lifecycle of an innovation and in view of the geographical context in which end users are located. More specifically, this framework’s 5 value domains emphasize the notion that RIH should: (*i*) increase our ability to meet collective needs while tackling health inequalities (population health value); (*ii*) provide an appropriate response to contemporary health system challenges (health system value); (*iii*) deliver not only high-performing products but also affordable ones (economic value); (*iv*) be aligned with business strategies through which an enterprise can provide more value not only to users and purchasers but also to society (organizational value); and (*v*) reduce as much as possible the negative environmental impacts of health innovations along their whole lifecycle (environmental value).

These value domains not only bring to the fore the products and processes of RIH, but also the organizations that develop innovations and make them available to end-users. It thus establishes explicit linkages with the various entrepreneurial organizations (eg, start-ups, small and medium size enterprises, large manufacturers, not-for-profit enterprises, non-governmental organizations, etc) that are behind the development, production and distribution of the innovation.

Despite the growing importance attached to RIH^17^ and to innovations that can better address population health needs and system-level challenges, there are currently no tools that can help to determine whether an innovation fulfills RIH requirements. To bridge this research and policy gap, we developed and validated an evidence-informed assessment RIH Tool, which was designed to capture the upstream characteristics of a broad range of health innovations. While the Tool may be applied to assess mature technologies, it aims first and foremost to inform decisions made at an early stage in the development process, where “early” is understood in relation to the transformational impact the Tool may have over the innovation. As further explained throughout the article, such impact could entail redefining its development processes, its characteristics as a product and/or the characteristics of the organization that makes it available to end users.

The aim of this paper is thus threefold. We present the findings of an inter-rater reliability study of the RIH Tool, followed by findings from its application to a large sample of innovations (n = 25). Since this study brings to completion the tool development process, this paper also makes available the final version of the RIH Tool (see Supplementary file 1).

### Premises and Development Process of the Responsible Innovation in Health Tool

Five premises underlie the RIH Tool and conditioned our stepwise efforts to develop its constructs and measures. Firstly, the RIH Tool adopts a population health perspective. Though an innovation that provides individual health benefits is valuable, RIH should first and foremost increase our ability to attend to collective needs.^3^

Secondly, the overall responsibility of a given innovation is intimately linked to its context of use. Here, the key assumption is that the degree of responsibility of an innovation is not context-free and must be appraised in view of its broader context of use, which includes infrastructures, personnel, ethical and legal frameworks, access to care and social services, and financial, geographical and/or cultural barriers. This premise should structure the whole assessment process, linking the degree of responsibility of an innovation to “where in the world” it is going to be used and disseminated and that may be approached at the municipal, regional, provincial or national level.

Thirdly, the RIH Tool is meant to be used when an innovation can be made available for use in the regions where its intended users are located. A number of aspects may still be unknown, but effectiveness and safety studies are more likely to have been conducted.^6,12^

Fourthly, while the RIH Tool was not designed to assess an innovation against a standard option, it generates an overall responsibility score that may be used to compare the respective value of different innovations.

Finally, the RIH Tool is an evidence-informed tool and it must be applied by individuals who possess research skills and are able to retrieve and critically read scientific literature. In this regard, requirements for an appropriate application of the Tool include holding formal training in an applied discipline with a focus in health and social care as well as experience working within an interdisciplinary research team, and having access to relevant bibliographic databases and search engines for retrieving scientific peer-reviewed journals. After having retrieved and compiled the relevant sources of information, consensus over each criterion and attribute should be sought through deliberation.^14^ While the Tool is meant to be *applied* by researchers, its results are meant to be *used* by various health innovation stakeholders (eg, research funding agencies, technology transfer offices [TTOs], innovators, investors, etc). Since the latter do not typically hold research skills, they will have to rely on scholars who can apply the Tool.

The RIH Tool was developed through an iterative process wherein conceptual and empirical developments alternated. We reviewed the scientific literature on RRI to identify the key concepts, dimensions and indicators that could apply to RIH. We performed a web-based horizon scanning to identify a large set of health innovations with responsibility features. Results of this exercise^15^ plus a review of the international health system literature and bodies of knowledge that are relevant to RIH enabled our team to circumscribe the dimensions of RIH and develop preliminary versions of the RIH Tool. We relied on scholarships that are specific to health (eg, health technology assessment [HTA], ethical, legal, and social issues [ELSIs], determinants of health, health economics, health services research, etc) and others that are specific to technology-based entrepreneurship (eg, business models, frugal or “bottom of the pyramid” innovation, etc). We gathered feedback from health innovation experts and pre-tested 2 different versions of the tool (see Acknowledgements). To critically appraise and improve constructs validity, we performed a 2-round Delphi exercise with international experts in RRI, HTA, biomedical engineering, and bioethics. Findings of this Delphi study^18^ confirmed the importance, clarity and appropriateness of the criteria, attributes and scales we had developed and which are the object of the current study.

### Application of the Responsible Innovation in Health Tool

**Figure 1:**
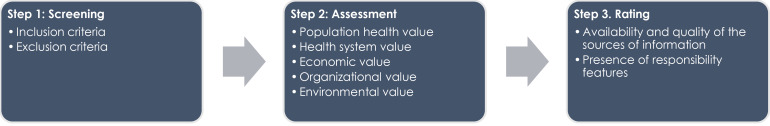
The 3-Step Process of the Tool’s Application.

The application of the RIH Tool follows a 3-step process: screening, assessment and rating (see Figure 1). The types of evidence sources that can be used to assess each criterion and attribute are indicated in the full version of the RIH Tool (see Supplementary file 1), which provides a simple classification of their quality level.

The *screening step* swiftly ascertains whether an innovation may potentially qualify as responsible innovations through 4 dichotomous inclusion and exclusion criteria. The inclusion criteria – *Determinants of health* and *Innovativeness* – are meant to select novel solutions that safely and effectively address at least one determinant of health. One important premise here is that if an innovation’s effectiveness and safety have not yet been demonstrated, there is little point in applying the RIH Tool. Likewise, the exclusion criteria – *Unavailability* and *Corporate social irresponsibility* – exclude from the assessment process innovations that are not available to intended users or that are produced by organizations that fail to behave responsibly.

The *assessment step* measures the presence of responsibility features through 9 attributes, which rely on a 4-level ordinal Likert-type scale, where:

- A implies a high degree of responsibility (5 points);
- B a moderate degree of responsibility (4 points);
- C a low degree of responsibility (2 points);
- D no particular signs of responsibility (1 point).

The attributes are organized within the 5 value domains of the RIH framework by Silva et al.^16^ The population health value domain comprises 3 attributes that capture the importance of the health needs addressed by the innovation (*Health relevance*), whether means to mitigate its *ELSIs*are available and the extent to which it tackles *Health inequalities*. Likewise, the health system value domain relies on 3 attributes that establish the degree of stakeholder engagement in the design, development and pilot stages of the innovation (*Inclusiveness*), whether it provides solutions to address health systems challenges (*Responsiveness*) and the extent to which it reduces labour intensity while enabling the provision of safe and effective services (*Level and intensity of care*). The economic value domain includes one attribute to assess whether the innovation was designed and produced in order to deliver greater value to more people using fewer resources (*Frugality*). The organizational value domain comprises one attribute to identify whether the organization that produces the innovation seeks to provide more value to users, purchasers and society through its *Business model*. The environmental value domain relies on one attribute to measure the extent to which the negative environmental impacts of the innovation are mitigated throughout its lifecycle (*Eco-responsibility*).

The *rating step* sums up the assessment results through a scoring system comprised of 2 components. The first refers to the availability and the quality of the information sources used to score each attribute, whereas the second refers to the responsibility features of the innovation. If more than one type of information is used to score an attribute, the source of highest quality is reported as follows:

- Type 1. Low quality information (1 point): technical documentation made available by the organization that produces the innovation;
- Type 2. Moderate quality information (2 points): reports by multilateral organizations (eg, World Health Organization, Organisation for Economic Co-operation and Development), governments, regulatory agencies, certification bodies or independent not-for-profit organizations that monitor and report on human and labour rights, animal welfare and environmental regulation;
- Type 3. High quality information (3 points): peer-reviewed scientific articles and systematic reviews of the literature (including HTAs, Cochrane Reviews, etc).

A scorecard is used to report and interpret these measures (see Supplementary file 1; an Excel spreadsheet for filling up the scorecard is available upon request). The overall responsibility features score establishes the mean score for all attributes and relies on a 4-level interval rating according to which “almost all,” “many,” “few” or “almost no RIH features are present.” The logic behind this benchmark scale follows the 4-level Likert-type scale used to assess the degree of responsibility for each attribute (ranging from A to D). The overall RIH score is considered meaningful only if the assessment relies on (*a*) a sufficient number of documented attributes (≥7/9 attributes) and (*b*) information sources of superior quality (mean score ≥2 points).

Table 1 summarizes the criteria, attributes and scales of the RIH Tool, which are organized around the 3-step application process explained above.

**Table 1:** An Overview of the RIH Tool Components

**Step**	**Definition of the Criterion or Attribute**	Type of Scale*
**Screening**		*Dichotomous (yes, no)*
Inclusion	Determinants of health: Factors inside and outside the health system that determine health across one’s life course.	Yes (include), No
	Innovativeness: Degree of novelty of the innovation, which may entail solving a problem in a novel way, combining novel components, materials or social interventions, or new processes of production, distribution, commercialization or delivery.	Yes (include), No
Exclusion	Unavailability: Innovation not available in the form of a ready-to-use product, process or system in the geographical region where its intended users are located.	No, Yes unavailable (exclude)
	Corporate social irresponsibility: Corporate actions, be they legal or illegal, that can harm people, animals or the environment.	No, Yes (exclude)
**Assessment**		*Ordinal (1 point*→* 5 points)*
Population health value	Health relevance**: Importance of the health needs addressed by the innovation within the overall burden of disease, considering the causes of death, injury and disability and associated risk factors in the region where the intended users are located.	The bottom quarter of all causes or risk factors (the lowest 25%) → The top quarter (75% and above)
	ELSIs: Means by which the negative impacts of the innovation on the moral and sociocultural well-being of individuals and groups and the legal and regulatory issues it raises can be mitigated.	None of the applicable ELSIs → Nearly all applicable ELSIs
	Health inequalities: Extent to which the innovation contributes to the reduction (or increase) of avoidable health status differences across individuals and groups that are associated with one’s socioeconomic status, social position and capabilities.	Increases inequalities → Reduces inequalities
Health system value	Inclusiveness: Degree of stakeholder engagement in the design, development and pilot stages of an innovation using an accountable method.	Did not engage stakeholders → Engaged a diverse and relevant set of stakeholders through a formal method and explained how their input was integrated in the design process
	Responsiveness: Ability to provide dynamic solutions to existing and emerging challenges in health systems (eg, demographic or epidemiologic shifts, service delivery or governance gaps).	No specific system-level challenges → A system-level challenge that is documented as being of high importance in the target region
	Level and intensity of care: Labour intensity optimization by mobilizing the most decentralized unit in the health system to provide the service when it is possible to do so effectively and safely.	Health and social care providers operating at the most specialized level of care within the health system → The patient, an informal caregiver or a health and social care provider operating in a non-clinical environment
Economic value	Frugality: Provision of greater value to more people by using fewer resources, which may entail: (*i*) affordability; (*ii*) focus on core functionalities and ease of use; and (*iii*) optimized performance.	No characteristics of frugal innovation → All 3 characteristics of frugal innovation
Organizational value	Business model: Organizational propensity to provide more value to users, purchasers and society through a business model that supports: (*i*) a social, not-for-profit and/or environmental mission; (*ii*) a freely usable or exploitable innovation; (*iii*) a redistributive pricing scheme; (*iv*) employees with particular needs; or (v) compliance with social responsibility programs.	None of the characteristics described → 3 of the characteristics described or more
Environmental value	Eco-responsibility: Reduction of negative environmental impacts along the innovation’s lifecycle stages: raw material sourcing; manufacturing; distribution; use; and disposal.	None of the key lifecycle stages → 3 key lifecycle stages or more
**Rating**	Availability of information: Number of attributes documented over the total number of attributes.	*Proportion threshold* Insufficient number of attributes (<7/9), Sufficient number of attributes (≥7/9)
	Quality of information sources: 3 types of source are hierarchized according to the expected level of objectivity in their reporting.	*Mean score threshold* Low to moderate quality (< 2), Moderate to high quality (≥2)
	Presence of RIH features: Overall measure of the innovation’s responsibility features.	*Ordinal interval* Almost no RIH features are present (1.0-2.0) à Almost all RIH features are present (4.1-5.0)

Abbreviations: ELSIs, ethical, legal, and social issues; RIH, responsible innovation in health. Source: Authors’ analysis of the Assessment Tool for Responsible Innovation in Health, 2019. Notes: (*) Further details about the definitions and scales can be found in the complete version of the RIH Tool provided in Supplementary Material. (**) To score the “Health relevance” attribute using the Global Burden of Disease Study data of the Institute of Health Metrics and Evaluation (Disability-Adjusted Life Years, 2017), an Excel spreadsheet is available upon request.

## Methods

As mentioned above, this study took place after having improved and validated the constructs of the RIH Tool with international experts in RRI, biomedical engineering, HTA and bioethics.^18^ We explain below how we assessed its reliably by measuring the extent to which 2 raters agree when applying the Tool to the same innovations and its applicability to a diversified sample of innovations.

### Study Design and Data

Reliability studies are widely used in health and social sciences to estimate whether measurement tools provide stable or consistent responses.^19^ Inter-rater reliability refers to the extent to which classifications of the same set of objects performed by 2 or more raters coincide.^20^ Our study design was meant to measure the extent to which 2 raters agree when they apply the RIH Tool to the same set of health innovations (the objects).

We conducted an inter-rater reliability assessment in early 2019. Following Gwet’s recommendations regarding the optimal number of objects required to achieve a sufficient level of accuracy and minimize the “standard error associated with the percent agreement (*p*_a_) between 2 arbitrary raters,”^20^ we set an error margin of ±0.20 to estimate our sample size. The latter included 25 health innovations.

We used a non-probabilistic sampling method for both raters and objects. Two co-authors (AAL, RRO) were chosen as raters since their profile is similar to that required by the application of the RIH Tool. We prioritized innovations that possessed some of the responsibility features identified by Silva and colleagues’ framework^16^ and sought to diversify the sample as much as possible by including different types of health innovation (eg, diagnostic tests, medical devices, digital solutions, mobile care units, etc). Because we wanted to examine whether the Tool could differentiate RIH from more traditional innovations, we also included 3 innovations with no particular signs of responsibility features.

Information from multiple sources was collected by the 2 raters for the whole sample. Excerpts that were specifically relevant to each criterion and attribute were tabulated in an Excel spreadsheet. When information was impossible to identify through electronic databases, we indicated “information not available.” All information excerpts were cross-checked by the first author to ensure they were clear and relevant to answer the questions of the RIH Tool. Using the evidence synthesized in the Excel spreadsheet, the RIH Tool was applied independently by each rater. An Excel version of the scorecard was used to report ratings and generate the study’s database. Once inter-rater reliability results were calculated, a meeting was held to reach consensus.

### Study Measures

To establish inter-rater reliability, 2 categories of index are described in the literature: non-adjusted indices, such as the percent agreement (*p*_a_), and chance-adjusted indices (eg, Cohen’s kappa, Gwet’s AC, Scott’s Pi, Krippendorff’s alpha and Brenn-Prediger). All indices consider that 1 represents maximum reliability, while 0 indicates no reliability.

While chance-adjusted indices refer to the same concept of inter-rater reliability, they produce different results for the same agreements because paradoxes and abnormalities affect their chance agreement.^21^ Some indices, such as Cohen’s kappa, are exposed to severe paradoxes and produce coefficients that are unexpectedly low when compared to the percent agreement, while others – Gwet’s AC, Brenn-Prediger – are more paradox-resistant.^20,22-24^

We thus selected 3 reliability measures: a non-adjusted index (percent agreement), a more paradox-resistant chance-adjusted index (Gwet’s AC) and a correlation coefficient to estimate the strength and direction of the linear relationship between 2 continuous variables (Pearson’s *r*). We used the latter “to evaluate the extent to which ratings from 2 raters are related.”^20^

### Data Analysis

We used unweighted coefficients for nominal ratings (the Tool’s screening criteria) and weighted coefficients for ordinal ratings (its assessment attributes). Quadratic weights were used to calculate weighted coefficients. We applied the Landis and Koch’s kappa benchmark scale^25^ to interpret the strength of the agreement coefficient: “Poor” (<0.0); “Slight” (0.0 to 0.20); “Fair” (0.21 to 0.40); “Moderate” (0.41 to 0.60); “Substantial” (0.61 to 0.80); and “Almost perfect” (0.81 to 1.00).

We conducted the recommended steps to check whether we could use the Pearson’s correlation coefficient.^20^ Since all conditions were met, we applied the “rule of thumb for interpreting the size of a correlation coefficient,”^26^ ranging from “negligible correlation” (0.00 to 0.30 or 0.00 to -0.30) to “very high positive or negative correlation” (0.90 to 1.00 or -0.90 to -1.00). Finally, standard errors, 95% confidence intervals and *P*values associated to each coefficient were calculated.

## Results

### Reliability

Table 2 shows inter-rater agreement results for all screening criteria and assessment attributes once the RIH Tool was applied by 2 independent raters to the sample of 25 innovations. The percent agreement is 100% for all inclusion and exclusion criteria as well as for the screening outcome (ie, the decision to proceed to assessment or not). Random chance is zero and the chance-adjusted coefficient is 1, which is the maximum possible value.

**Table 2:** Inter-rater Agreement Results

	**N**	**Percent Agreement**	Coefficient*	**Standard Error**	**95% CI**	***P *** **Value**
Screening criteria						
Determinants of health	25	100%	1	0	1 to 1	n/a
Innovativeness	25	100%	1	0	1 to 1	n/a
Unavailability	25	100%	1	0	1 to 1	n/a
Corporate social irresponsibility	25	100%	1	0	1 to 1	n/a
*Screening outcome*	25	100%	1	0	1 to 1	n/a
Assessment attributes						
Health relevance	25	100%	1	0.00000	1 to 1	n/a
ELSIs	22	92%	0.812	0.06476	0.677 to 0.946	<.001
Health inequalities	20	84%	0.630	0.17706	0.259 to 1	.002
Inclusiveness	22	90%	0.741	0.08779	0.558 to 0.923	<.001
Responsiveness	22	93%	0.903	0.07371	0.750 to 1	<.001
Level and intensity of care	23	92%	0.827	0.11283	0.593 to 1	<.001
Frugality	22	98%	0.964	0.01933	0.924 to 1	<.001
Business model	21	97%	0.888	0.03879	0.807 to 0.969	<.001
Eco-responsibility	12	94%	0.855	0.09243	0.652 to 1	<.001
Overall RIH features score	25	97%	0.919	0.02631	0.864 to 0.973	<.001

Abbreviations: ELSIs, ethical, legal, and social issues; RIH, responsible innovation in health. Source: Authors’ analysis of the Assessment Tool for Responsible Innovation in Health, 2019. Note: (*) The Gwet’s AC1 is shown for the nominal ratings of the screening criteria (yes, no). The Gwet’s AC2 is shown for the ordinal ratings of the assessment attributes (A, B, C, D) and for the interval ratings of the overall RIH features score (4.1-5.0; 3.1-4.0; 2.1-3.0; 1.0-2.0).

For the assessment attributes, percent agreement ranges from 84% for *Health inequalities* to 100% for *Health relevance*. For 7 attributes, the Gwet’s coefficients are higher than 0.81, which is interpreted as “almost perfect” according to Landis and Koch.^25^ For the other 2 attributes, reliability is “substantial”: *Health Inequalities* (0.630) and *Inclusiveness* (0.741).

A percent agreement of 97% and a Gwet’s coefficient of 0.919 (95% CI: 0.864 to 0.973) were obtained for the *Overall RIH features score*. This suggests an “almost perfect” agreement between raters. Since all *P*values are ≤.01, inter-rater agreement measures are statistically significant.

**Figure 2:**
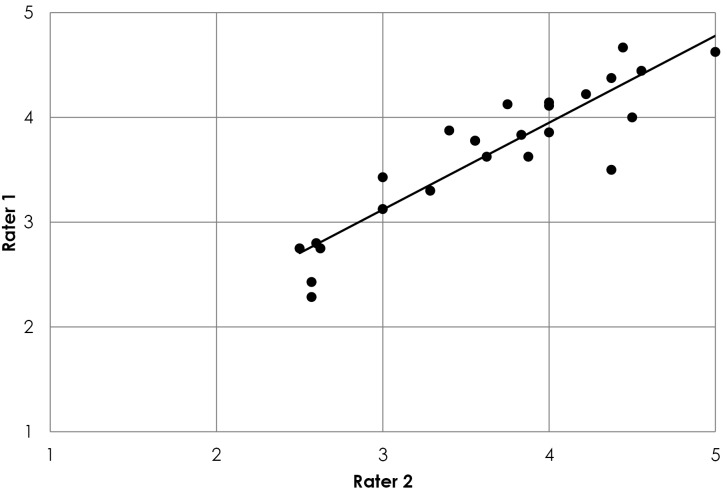
Overall RIH Features Scores by Rater 1 and Rater 2. Abbreviation: RIH, responsible innovation in health.

The Pearson’s moment correlation coefficient for the *Overall RIH features score* is 0.909 (*P* ≤ .01) which indicates a very high positive correlation. A visual analysis of this correlation can be seen on Figure 2, which shows the overall RIH features scores obtained by Rater 1 and Rater 2 for the whole sample of innovations.

### Results From the Application of the Tool

Table 3 shows the results of the application of the RIH Tool to the whole sample of health innovations. At the screening step, 19 innovations met all the inclusion and exclusion criteria and 6 did not. The latter were either not available in the geographical regions where intended users are located (n = 3) or the organization that produces the innovation had been or was currently involved in irresponsible corporate actions (n = 3). In principle, these innovations would be excluded from further assessment, but for the purpose of this paper the whole sample was assessed.

**Table 3:** Results of the Application of the RIH Tool to the Whole Sample of Innovations (n = 25)

**Screening Step**	**Include**	**Exclude**		
Determinants of health	25 (100%)	0 (0%)		
Innovativeness	25 (100%)	0 (0%)		
Unavailability	22 (88%)	3 (12%)		
Corporate social irresponsibility	22 (88%)	3 (12%)		
*Screening outcome: Proceed to assessment?*	**Yes**	**No**		
	19 (76%)	6 (24%)		
**Assessment step**	**A**	**B**	**C**	**D**
Health relevance	21 (84%)	1 (4%)	1 (4%)	2 (8%)
ELSIs	1 (5%)	4 (18%)	16 (73%)	1 (5%)
Health inequalities	10 (50%)	6 (30%)	2 (10%)	2 (10%)
Inclusiveness	2 (10%)	4 (19%)	13 (62%)	2 (10%)
Responsiveness	18 (82%)	2 (9%)	1 (5%)	1 (5%)
Level and intensity of care	15 (65%)	2 (9%)	5 (22%)	1 (4%)
Frugality	11 (50%)	8 (36%)	3 (14%)	0 (0%)
Business model	7 (33%)	5 (24%)	5 (24%)	4 (19%)
Eco-responsibility	3 (27%)	1 (9%)	7 (64%)	0 (0%)
**Rating step**				
Number of attributes documented	≥ 7/9	< 7/9		
	22 (88%)	3 (12%)		
Mean score of information sources quality	≥ 2	< 2		
	9 (36%)	16 (64%)		
Overall RIH mean score	4.1-5.0	3.1-4.0	2.1-3.0	1.0-2.0
	6 (24%)	13 (52%)	6 (24%)	0 (0%)

Abbreviations: ELSIs, ethical, legal, and social issues; RIH, responsible innovation in health. Source: Authors’ analysis of the Assessment Tool for Responsible Innovation in Health, 2019. Note: Although 6 innovations did not meet the inclusion and exclusion criteria and therefore would not be considered for further assessment, all 25 innovations in the sample were assessed in order to fulfill the purpose of the inter-rater reliability exercise.

A large majority of the 25 innovations obtained the highest score (A) for 3 attributes: *Health relevance* (84%), *Responsiveness*(82%) and *Level and intensity of care* (65%). Half of the sample obtained the highest score for *Health inequalities* (50%) and *Frugality* (50%) and close to a third obtained a B score for the same attributes (respectively, 30% and 36%). While a low score (C) was predominantly obtained for 3 attributes — *ELSIs* (73%), *Inclusiveness* (62%) and *Eco-responsibility* (64%) —, only a small portion of the sample (ranging from 4% to 19%) obtained the lowest score (D) and this was observed for 7 attributes.

At the rating step, 88% of the innovations had a sufficient number of attributes documented (≥7/9), whereas the assessment of 12% of the sample was compromised by missing information (<7/9). For 36% of the innovations, the assessment was based on sources of moderate to high quality (mean score ≥2 points), while for 64% of the sample, it was compromised by sources of low to moderate quality (<2 points).

The overall RIH mean score fell within the highest interval for 24% of the innovations (*Almost all RIH features are present*), within the second interval for 52% of them (*Many RIH features are present*) and within the third interval for 24% of the sample (*Few RIH features are present*). We observed that the more traditional innovations obtained lower scores, but none of the 25 innovations fell into the lowest interval (*Almost no RIH features are present*).

### Interpretation of the Overall Score

To illustrate how the results of the RIH Tool could inform decisions by highlighting the strengths and weaknesses of various innovations, Table 4 shows the breakdown of the assessment for 3 innovations that obtained different overall RIH features scores as well as the mean score, standard deviation and minimum and maximum values for the sample as a whole.

**Table 4:** An Illustration of the Outputs of RIH Tool

**Assessment Attributes**	**Innovation 1**	**Innovation 2**	**Innovation 3**	**Sample (n = 25)**	
				**Mean**	**SD**	**Min**	**Max**
Population health value							
Health relevance	4	5	5	4.5	1.2	1	5
ELSIs	4	2	1	2.5	1.0	1	5
Health inequalities	5	5	-	4.0	1.4	1	5
Health system value							
Inclusiveness	4	2	2	2.6	1.2	1	5
Responsiveness	5	5	4	4.6	1.1	1	5
Level of care	5	5	1	4.1	1.4	1	5
Economic value							
Frugality	5	2	-	4.2	1.0	2	5
Organizational value							
Business model	4	4	1	3.3	1.6	1	5
Environmental value							
Eco-responsibility	5	2	2	3.0	1.4	2	5
**Overall RIH score**	4.6	3.6	2.3	3.7	1.5	2.3	4.8
	Almost all RIH features are present	Many RIH features are present	Few RIH features are present				

Abbreviations: ELSIs, ethical, legal, and social issues; RIH, responsible innovation in health; SD, standard deviation. Source: Authors’ analysis of the Assessment Tool for Responsible Innovation in Health, 2019. Note: A= 5 points; B = 4 points; C = 2 points; D = 1 point.

Innovation 1, a menstrual cup distributed for free to users in developing countries through a “buy one, give one” model, obtained A or B scores for all 9 attributes, explaining why it fell into the highest interval. Innovation 2 is a set of tools aimed to scan, design and print 3D upper limb prosthetics for children and it fell within the second highest interval by obtaining high scores for 5 out of 9 attributes. In contrast, Innovation 3, an implantable cardioverter-defibrillator which is imaging device-friendly, obtained high scores for *Health relevance* and *Responsiveness*, but C or D scores for 5 attributes. Since no more than 2 attributes were not assessed because information was missing — *Health inequalities* and *Frugality*—, the overall RIH features score for this innovation (2.3) is considered meaningful.

## Discussion

While previous research confirmed the face validity of the RIH Tool’s constructs,^18^ this study adds to current knowledge by showing that the RIH Tool is both reliable and applicable to a diversified set of health innovations.

On the one hand, we found complete agreement between the 2 raters for the 4 screening criteria, an “almost perfect” agreement for 7 assessment attributes and a “substantial” agreement for 2 attributes. An “almost perfect” agreement was observed for the Overall RIH features score and all measures were statistically significant. On the other hand, findings from the RIH Tool’s application show that 50% of the sample or more obtained the A score for *Health relevance*, *Health inequalities*, *Responsiveness*, *Level and intensity of care*, and *Frugality*. This suggests that those who develop RIH seek to: (*i*) tackle a burden of disease falling within the top quartile; (*ii*) cater to the needs of vulnerable groups that are not met by current solutions; (*iii*) respond to system-level challenges that are considered of high importance in the target region; (*iv*) support patients and caregivers who operate in a non-clinical setting; and (*v*) reduce substantially the costs of production and use of an innovation by focusing on the core functionalities its users require and optimizing its performance level. More than half of the sample obtained a C score for *ELSIs*, *Inclusiveness* and *Eco-responsibility*. This emphasizes the obstacles innovators face when seeking to: (*i*) match their innovation with proper means to mitigate ethical, legal and social issues; (*ii*) engage a diverse and relevant set of stakeholders in the development process through an accountable method; and (*iii*) integrate eco-responsibility concerns at key stages in their innovation’s lifecycle.

### Study Limitations

These findings should be appraised considering our study’s strengths and weaknesses. With regards to the inter-rater reliability assessment, 4 limitations must be kept in mind. First, less than 25 innovations of our sample were fully rated because information was lacking for a number of attributes. Since levels of accuracy are affected by the sample size, the accuracy of our reliability measures may vary.^20^ Second, objects and raters were not randomly selected, which limits our ability to draw inferences regarding its application in a real-world context, that is, when applied by raters who may not be as familiar with the RIH framework and Tool as were our study raters. Third, even though Gwet’s agreement coefficient is considered a more paradox-resistant index than alternative chance-adjusted ones, it tends to deliver more liberal estimates of reliability when compared to other indices.^21^ Fourth, ratings may have been affected by information asymmetry between raters. Because they were responsible for documenting different innovations, they were not equally exposed to the dataset and their judgements may have been influenced by the broader knowledge acquired when documenting each innovation.

Though the application process of the RIH Tool is relatively straightforward, finding appropriate evidence to document all criteria and attributes may prove challenging. Determining whether an innovation effectively and safely address one or more determinants of health is a delicate task since few effectiveness and safety studies may have been published by the time the RIH Tool is applied, especially when regulatory approvals are not required. The RIH Tool user guide, which is under development, will indeed clarify how appropriate thresholds can be operationalized when uncertainty is not too high (see also further details below). The corporate social irresponsibility exclusion criterion requires searching for legitimate public statements describing infringements in one domain of irresponsible corporate actions. It is important to refer to specific governmental agencies, regulatory bodies or independent not-for-profit organizations that monitor human and labour rights, animal welfare and environmental regulations. In this study, information relevant to the Eco-responsibility attribute was available for only 44% of the sample. This is a reflection of the growing, yet limited literature on the environmental impacts of health technologies.^27,28^ Within this perspective, one should acknowledge that the RIH Tool reflects the level of responsibility of an innovation at a given point in time. Since characteristics of the development process, of the innovation itself and of the organization that produces it may change over time, cross-sectional assessments may produce different results depending on when they are performed. In other words, the quantity and scope of information available to apply the RIH Tool are likely to change over time and affect the overall score an innovation obtains.

Despite these limitations, the RIH Tool brings a novel and important contribution to current knowledge and avoids some of the limitations associated to the use of MCDA in HTA processes. According to the current literature,^6,29,30^ several MCDA criteria overlap and are not independent; the weight that can be set for each criterion downplays the trade-offs that will be made between different value domains; the deliberative process underlying MCDA was criticized since it supports a form of economic evaluation based on stakeholders’ preferences; and deliberations may unduly be replaced by decision algorithms that reinforce a deterministic approach to decision-making.

In view of these criticisms, the strengths of the RIH Tool may be summarized as follows. First, its criteria and attributes were developed and revised through a stepwise and empirically grounded process to eliminate any overlaps and consolidate their independence. Second, since all 9 attributes are considered equally important and thus have an equal weight in the overall RIH score, the trade-offs they entail will remain explicit and will have to be reported in the scorecard. Choosing to do otherwise and opening up the possibility for raters and decision-makers to assign weights to the different attributes would have necessitated strong evidence that there are attributes that can be justified as being more important than others. We have not encountered such arguments in the bodies of literature we reviewed and thus gave an equal weight to all attributes. Yet, aligned with the evidence stressing the need for RIH to support equity and sustainability in health systems, 2 value dimensions (population health and health system) possess 3 attributes each, which emphasizes their role in the overall RIH score. Third, while the outcomes of the RIH Tool are not dependent upon stakeholders’ preferences, it is meant to complement cost-effectiveness analyses since its Frugality attribute foregrounds characteristics that are not typically considered in traditional economic evaluations that emphasize a cost-benefit logic.^31^ Finally, the overall RIH score supported by the Tool cannot be established without a deliberation between at least 2 applied scientists who are required to review the evidence that justifies the score for each attribute and that is reported in the scorecard. Hence, the overall RIH score cannot be derived by a single individual or without due consideration of the context in which the innovation is used.

### Policy Implications

Keeping in mind the distinction between those who apply the Tool (applied scientists) and those who use its results to inform upstream decisions (eg, investors, innovation developers, incubators, health innovation policy-makers, etc), we believe that the policy implications of the RIH Tool are threefold. First, it supports an evidence-informed judgment that makes it possible to differentiate, at an early stage, innovations that possess key responsibility features from innovations with no particular signs of responsibility. While the latter are not to be considered “irresponsible,” the former are by design more likely to generate greater social, economic and environmental value. Recognising that the time and efforts needed to develop innovative health and social care services, medical devices or information technology-based solutions differ considerably, we consider that an assessment of the degree of responsibility of an innovation would arrive “too late” when it can no longer transform what the innovation will be able to achieve in practice.

Second, the RIH Tool comes with a conservative and explicit rule for the interpretation of its overall score. One must consider whether the assessment relies on a sufficient number of attributes and on information sources of superior quality. When one of these 2 requirements is not met, the overall score is not considered meaningful and should not be used to inform decisions. Our findings show that the number of attributes documented was considered sufficient for 88% of the innovations, but assessments were compromised by sources of inferior quality for 64% of the sample. It is thus important to take these 2 components into account before making a judgment. Furthermore, when there are no information sources available at all, the Tool cannot be applied to establish an overall RIH score, but its attributes and their corresponding scales provide sufficiently concrete elements to inform upstream decisions. For instance, the “eco-responsibility” attribute stresses the importance of attending to environmental concerns at more than one key stage in an innovation’s lifecycle. This information has direct relevance to innovation stakeholders at an early stage. We thus presume that the Tool remains informative even when there’s a lack of good quality studies to fully perform an assessment.

Third, the RIH Tool was designed to support decisions in a variety of settings that affect the emergence but also the adoption of innovations. For instance, it may be used by university-based TTOs, technology developers, investors and philanthropic foundations to better align innovation development processes with RIH. It provides health innovation policy-makers with operational definitions of value domains, criteria and attributes that can inform calls for oriented-research proposals, a practice adopted by many agencies concerned with the integrity and societal benefits of the research they support.^32^ If applied downstream to guide the adoption of more mature technologies, the RIH Tool could support a value-based approach in procurement processes that seek to maximize value by considering economic and environmental impacts, social preferences and suppliers’ business practices.^33,34^

### Further Research

All of these likely applications of the RIH Tool are not without tensions and thus form important areas for further research.^17^ It is particularly important to examine the application and outcomes of the RIH Tool in a real-world setting. While the current study was designed to establish whether the Tool perform well (ie, show consistent results) when it is applied under the right conditions, knowing whether similar results are observed when the Tool is applied by raters who are less familiar with its attributes and scales is important. Since our findings indicate that the level of agreement was slightly lower for 2 attributes —*Health inequalities* and *Inclusiveness*—, their application deserve particular attention. To address such issues, a user guide and a webinar will be developed to support the application of the Tool. We assume that raters should be familiar with the RIH framework^16^ and rely on the user guide when first applying the Tool. They should also hold formal training in an applied discipline relevant to health innovations and experience working within an interdisciplinary research team since consensus should be reached over all criteria and attributes. Further research could thus identify how to best interpret ambiguous or controversial evidence and establish a clear and accountable deliberative process for consensus making.

It would also be important to study decision-makers’ readiness to use the outputs of the RIH Tool and whether understanding and perceived usefulness of its key dimensions vary across groups. For instance, in a recent study, we found that Canadian health innovators (in Quebec and Ontario) are supportive of several dimensions of the RIH framework and call for policies and regulations that can provide incentives to the design, production and commercialization of more responsible innovations.^35^ Within a similar train of thought, further studies could examine the views and practices of social finance investors, public health system purchasers and private foundations.

## Conclusion

Through a rigorous development process that combined conceptual and empirical analyses, the relevance, clarity and validity of the RIH Tool were systematically improved. By confirming key aspects of its reliability and applicability, this study brings its development to completion. It is now ready to be jointly put into action by scholars, health innovation policy-makers and other innovation stakeholders who wish to establish the degree of responsibility of an innovation along 9 attributes that are rarely if ever considered altogether in MCDA or HTA.

The future uptake of the RIH Tool by the broader health innovation community could steer the supply of health innovations towards solutions that consolidate our ability to address important population health needs, tackle health inequalities, provide timely responses to contemporary health system challenges, deliver high-performing and affordable technologies and reduce their negative environmental impacts.

## Acknowledgements

This article stems from the In Fieri research program (http://infieri.umontreal.ca) on RIH, which is led by the corresponding author. We would like to thank the Editor and 2 anonymous reviewers for their constructive criticisms. We are also grateful to those who contributed to the development of the RIH Tool: (*a*) members of our research team who provided us with insightful comments: Jeremy Bouchez, Geneviève Daudelin, Dominique Grimard, Renata Pozelli, Lysanne Rivard, Federico Roncarolo, and Patrick Vachon; (*b*) experts of our research program who participated to a full-day meeting in Montreal, Canada, on October 21, 2016: Érica Barbosa, Catherine Beaudry, Antoine Boivin, Jean-Louis Denis, Philippe Gauthier, Nicola Hagemeister, Sophie Méchin, Fiona Miller, Öner Tulum, and Alexis Wise; (*c*) graduate students who pre-tested early versions of the Tool in courses taught by Nicola Hagemeister at École de Technologie Supérieure (ETS) and by Pascale Lehoux at École de santé publique de l’Université de Montréal (ESPUM); and (*d*) international experts who criticized 2 earlier versions of the RIH Tool through a Delphi process and provided us with invaluable comments.

## Ethical issues

The Research Ethics Review Board of the University of Montreal approved the empirical studies that led to the development of the RIH Tool (CERES-D #17-024).

## Competing interests

Authors declare that they have no competing interests.

## Authors’ contributions

HPS and PL contributed to the conception and design of the study. HPS wrote the first draft of the manuscript and PL contributed to its subsequent iterations. PL obtained funding, contributed important intellectual content, and supervised the study. AAL and RRO were responsible for collecting data and performing the inter-rater reliability assessment. HPS conducted most data analyses. All authors contributed to the interpretation of the literature and findings and revised critically preliminary versions of the RIH Tool. All authors read and approved the final version.

## Authors’ affiliations

^1^Public Health Research Institute, University of Montreal, Montreal, QC, Canada. ^2^School of Public Health, University of Montreal, Montreal, QC, Canada.

## Funding

This research was funded by an operating grant from the Canadian Institutes of Health Research (CIHR; #FDN-143294). Our research group infrastructure is supported by the Fonds de la recherche en santé du Québec (FRQ-S).

## Supplementary files

Supplementary file 1. The In Fieri Assessment Tool for RIH.

## Key Messages

**Implications for policy makers**

- The purposes, functions, and costs of innovations should be examined before they make their way into health systems.
- The responsible innovation in health (RIH) Tool directs policy-makers’ attention “upstream,” where they can foster innovations that can tackle significant system-level challenges and support more equitable and sustainable health services.
- A wide range of health innovation stakeholders, including Technology Transfer Offices (TTOs), investors and philanthropic foundations may rely on its results to identify innovations that possess key responsibility features.
- The RIH tool is both reliable and applicable to a wide set of innovations, but the interpretation of its results must consider whether the assessment relies on a sufficient number of attributes and on information sources of superior quality.

**Implications for public**

The way new health technologies are being designed, produced and brought to market raises significant economic, ethical and social issues. We developed a tool that can inform the development of innovations that support more equitable and sustainable healthcare. The tool takes into consideration several attributes, including health inequalities, inclusiveness, frugality and eco-responsibility.

## References

[1] Fineberg HV (2012). Shattuck Lecture A successful and sustainable health system--how to get there from here. N Engl J Med.

[2] Garber S, Gates SM, Keeler EB (2014). Redirecting innovation in US health care: options to decrease spending and increase value. Rand Health Q.

[3] Lehoux P, Roncarolo F, Silva HP, Boivin A, Denis JL, Hebert R (2019). What health system challenges should responsible innovation in health address? insights from an international scoping review. Int J Health Policy Manag.

[4] Macdonnell M, Darzi A (2013). A key to slower health spending growth worldwide will be unlocking innovation to reduce the labor-intensity of care. Health Aff (Millwood).

[5] Tony M, Wagner M, Khoury H (2011). Bridging health technology assessment (HTA) with multicriteria decision analyses (MCDA): field testing of the EVIDEM framework for coverage decisions by a public payer in Canada. BMC Health Serv Res.

[6] Marsh KD, Sculpher M, Caro JJ, Tervonen T (2018). The use of MCDA in HTA: great potential, but more effort needed. Value Health.

[7] Marsh K, Lanitis T, Neasham D, Orfanos P, Caro J (2014). Assessing the value of healthcare interventions using multi-criteria decision analysis: a review of the literature. Pharmacoeconomics.

[8] Marsh K, Goetghebeur M, Thokala P, Baltussen R. Multi-Criteria Decision Analysis to Support Healthcare Decisions. London, UK: Springer; 2017.

[9] Adunlin G, Diaby V, Xiao H (2015). Application of multicriteria decision analysis in health care: a systematic review and bibliometric analysis. Health Expect.

[10] Diaby V, Campbell K, Goeree R (2013). Multi-criteria decision analysis (MCDA) in health care: a bibliometric analysis. Oper Res Health Care.

[11] Goetghebeur MM, Cellier MS (2018). Can reflective multicriteria be the new paradigm for healthcare decision-making? the EVIDEM journey. Cost Eff Resour Alloc.

[12] Wahlster P, Goetghebeur M, Kriza C, Niederlander C, Kolominsky-Rabas P (2015). Balancing costs and benefits at different stages of medical innovation: a systematic review of Multi-criteria decision analysis (MCDA). BMC Health Serv Res.

[13] von Schomberg R (2013). A Vision of responsible research and innovation.

[14] Stilgoe J, Owen R, Macnaghten P (2013). Developing a framework for responsible innovation. Res Policy.

[15] Lehoux P, Pacifico Silva H, Pozelli Sabio R, Roncarolo F (2018). The unexplored contribution of responsible innovation in health to Sustainable Development Goals. Sustainability.

[16] Pacifico Silva H, Lehoux P, Miller FA, Denis JL (2018). Introducing responsible innovation in health: a policy-oriented framework. Health Res Policy Syst.

[17] Stahl BC (2019). Who is responsible for responsible innovation? lessons from an investigation into responsible innovation in health: Comment on “What health system challenges should responsible innovation in health address? Insights from an international scoping review”. Int J Health Policy Manag.

[18] Pacifico Silva H, Lehoux P, Hagemeister N (2018). Developing a tool to assess responsibility in health innovation: Results from an international Delphi study. Health Policy Technol.

[19] Bolarinwa OA (2015). Principles and methods of validity and reliability testing of questionnaires used in social and health science researches. Niger Postgrad Med J.

[20] Gwet KL. Handbook of Inter-Rater Reliability. 4th ed. Advanced Analytics, LLC; 2014.

[21] Zhao X, Liu JS, Deng K (2013). Assumption behind intercoder reliability indices.

[22] Quarfoot D, Levine RA (2016). How robust are multirater interrater reliability indices to changes in frequency distribution?. Am Stat.

[23] Wongpakaran N, Wongpakaran T, Wedding D, Gwet KL (2013). A comparison of Cohen’s Kappa and Gwet’s AC1 when calculating inter-rater reliability coefficients: a study conducted with personality disorder samples. BMC Med Res Methodol.

[24] Feinstein AR, Cicchetti DV (1990). High agreement but low kappa: I The problems of two paradoxes. J Clin Epidemiol.

[25] Landis JR, Koch GG (1977). The measurement of observer agreement for categorical data. Biometrics.

[26] Hinkle DE, Wiersma W, Jurs SG. Applied Statistics for the Behavioral Sciences. Houghton Mifflin; 2003.

[27] Polisena J, De Angelis G, Kaunelis D, Gutierrez-Ibarluzea I (2018). Environmental impact assessment of a health technology: a scoping review. Int J Technol Assess Health Care.

[28] Campion N, Thiel CL, Woods NC, Swanzy L, Landis AE, Bilec MM (2015). Sustainable healthcare and environmental life-cycle impacts of disposable supplies: a focus on disposable custom packs. J Clean Prod.

[29] Garau M, Devlin NJ (2017). Using MCDA as a decision aid in health technology appraisal for coverage decisions: opportunities, challenges and unresolved questions.

[30] Culyer AJ. Deliberative Processes in Decisions about Health Care Technologies: Combining Different Types of Evidence, Values, Algorithms and People. OHE Briefing, No. 48, June 2009. https://ssrn.com/abstract=2640171.

[31] Weyrauch T, Herstatt C (2016). What is frugal innovation? three defining criteria. J Frugal Innov.

[32] Davis M, Laas K (2014). “Broader impacts” or “responsible research and innovation”? a comparison of two criteria for funding research in science and engineering. Sci Eng Ethics.

[33] Robinson JC (2015). Biomedical innovation in the era of health care spending constraints. Health Aff (Millwood).

[34] Miller FA, Lehoux P, Peacock S (2019). How procurement judges the value of medical technologies: a review of healthcare tenders. Int J Technol Assess Health Care.

[35] Rivard L, Lehoux P (2020). When desirability and feasibility go hand in hand: innovators’ perspectives on what is and is not responsible innovation in health. J Responsible Innov.

