# Supplementary material for: Fostering Responsible Innovation in Health: An EvidenceInformed Assessment Tool for Innovation Stakeholders
Source: Int J Health Policy Manag. 2020 Mar 15;10(4):181–91. doi: 10.34172/ijhpm.2020.34 (PMC8167270; doi:10.34172/ijhpm.2020.34)
Supplement: Supplementary file 1 — The In Fieri Assessment Tool for RIH. [file ijhpm-10-181-s001.pdf]

The In Fieri Assessment Tool for Responsible Innovation in Health (RIH)

© 2020 Silva, H.P., Lefebvre, A.-A., Oliveira, R.R., Lehoux, P.

Background

The development of this Tool was inspired by the Responsible Research and Innovation (RRI) scholarship that identifies four process requirements to develop innovations in a responsible way: 1) inclusion of diverse stakeholders; 2) anticipation of risks, impacts and unintended consequences; 3) reflexivity about the norms and values underlying innovations; and 4) responsiveness to unforeseen and undesirable changes.

RRI also seeks to align innovation with important economic, social or environmental challenges. Examples of such challenges include the Seven Grand Challenges of the European Community, The Global Grand Challenges and the Sustainable Development Goals of the United Nations.

The Responsible Innovation in Health (RIH) framework: Key references

The In Fieri Tool relies on the conceptual framework described in: Silva, H.P., Lehoux, P., Miller, F.A., Denis, J.-L., (2018). Introducing Responsible Innovation in Health: A policy-oriented framework. *Health Research Policy and Systems*. 16(1): 90. This article provides the references for the attributes and should be read before applying the Tool.

The method through which the Tool's constructs were developed is described in: Silva, H.P., Lehoux, P., Hagemeister, N. (2018). Developing a tool to assess responsibility in health innovation: Results from an international Delphi study. *Health Policy and Technology*. 7(4): 388-396.

Definition of RIH

RIH consists in a collaborative endeavour wherein stakeholders are committed to clarify and meet a set of ethical, economic, social and environmental principles, values and requirements when they design, finance, produce, distribute and use sociotechnical solutions to address the needs and challenges of health systems in a sustainable way. RIH refers to the innovation as well as to the organization that develops and makes it available to intended users. The principles, values and requirements of RIH are applied throughout a technology's lifecycle, promoting the best social and environmental practices.

Who should apply the Tool?

The Tool is meant to be applied by people who possess research skills and are able to retrieve and critically read scientific literature. Judgment over each criterion and attribute should be made by an interdisciplinary team after having searched, retrieved and compiled the relevant sources of information.

Who may use the results of the Tool and when?

The Tool was designed to inform decisions made at an early stage by innovators, investors, research funding agencies, Technology Transfer Offices (TTOs), etc. "Early" should be understood in relation to the transformational impact the Tool may have over the innovation. Such impact could entail redefining its characteristics at the process-, product- and/or organizational-level.

Premises of the Tool

Context of use of the innovation: The overall responsibility of a given innovation is intimately linked to how and where it is used. Hence, the Tool should be applied in view of the geographical regions where the intended users of an innovation are located.

Individual vs. collective benefits: Although an innovation that provides individual health benefits is valuable, RIH should first and foremost increase our ability to attend to collective needs and challenges.

Safety and effectiveness: The Tool is meant to be used after the prototyping or piloting stage, that is, when an innovation can be made available for use in the regions where its intended users are located. A number of aspects may still be unknown at this stage (e.g., reimbursement by third-party payers, prices, long-term effects, etc.), but effectiveness and safety studies are more likely to have been conducted. One premise of our screening criteria is that if an innovation's effectiveness and safety have not yet been demonstrated, there is little point in applying the Tool.

Comparisons: Although the overall score may help to compare different types of innovation, the Tool was not designed to score an innovation against a standard option because such an alternative may be hard to identify or may not exist.

An overview of the Tool

Figure 1 illustrates the three-step process underlying the application of the Tool:

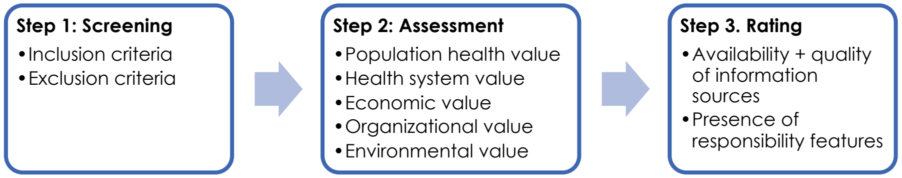

Figure 1. The three-step process

The **first step** quickly identifies whether an innovation may potentially qualify as a RIH through four dichotomous inclusion and exclusion criteria. The inclusion criteria are meant to select novel solutions that safely and effectively address a determinant of health. The exclusion criteria are meant to exclude from the assessment process innovations that are not available to intended users or that are produced by organizations involved in irresponsible corporate actions.

The **second step** assesses the presence of responsibility features through nine attributes organized into five value domains. For instance, the first two value domains — Population health and Health system — rely on three attributes each. All attributes rely on a four-level Likert-like scale, ranging from A to D, where A implies a high degree of responsibility and D implies no particular signs of responsibility.

The **third step** determines the outcomes of the assessment with the help of a scorecard (see Figure 2). The scoring system is comprised of two components. The first refers to the availability and the quality of the sources of information used to score each attribute. The second refers to the responsibility features of the innovation.

© 2020 Silva, H.P., Lefebvre, A.-A., Oliveira, R.R., Lehoux, P.

The scorecard

The scorecard below is used to report the sources of information upon which the assessment relies as well as two mean scores: 1) the information quality mean score; and 2) the overall responsibility features score. The interpretation of these measures is explained at the end of this document.

Reporting the information upon which the assessment relies

The Tool should be applied in a transparent and accountable way. Detailed extracts from the sources of information used by the raters that justify the score given to each attribute should be reported in the scorecard along with a list of references. To this end, an Excel version of the scorecard is available upon request (by sending an e-mail to the corresponding author: pascale.lehoux@umontreal.ca).

Quality of the sources of information

The types of information source that can be used to assess each criterion and attribute are indicated in the Tool and a simple classification for summarizing their quality is integrated in the scoring system. Because independent organizations and peer-reviewed publications are more likely to be objective in their reporting, they are classified as being of better quality for the Tool's assessment purposes.

- **Type 1. Low quality** (1 point): Technical documentation made available by the organization that produces the innovation.
- **Type 2. Moderate quality** (2 pts): Reports by multilateral organizations (e.g., WHO, OECD), governments, regulatory agencies, certification bodies or independent not-for-profit organizations that monitor and report on human and labour rights, animal welfare and environmental regulation.
- **Type 3. High quality** (3 pts): Peer-reviewed scientific articles and systematic reviews of the scientific literature (including Health Technology Assessments, Cochrane Reviews, etc.).

	Availability & Quality of information sources				Assessment of the attributes			
Value domains & Attributes	Available?	High 3 pts	Moderate 2 pts	Low 1 pt	A 5 pts	B 4 pts	C 2 pts	D 1 pt
**Population health value**								
1. Health relevance	☐	3	2	1	5	4	2	1
2. EL&S issues	☐	3	2	1	5	4	2	1
3. Health inequalities	☐	3	2	1	5	4	2	1
**Health system value**								
4. Inclusiveness	☐	3	2	1	5	4	2	1
5. Responsiveness	☐	3	2	1	5	4	2	1
6. Level & intensity of care	☐	3	2	1	5	4	2	1
**Economic value**								
7. Frugality	☐	3	2	1	5	4	2	1
**Organisational value**								
8. Business model	☐	3	2	1	5	4	2	1
**Environmental value**								
9. Eco-responsibility	☐	3	2	1	5	4	2	1

**Number of attributes documented:**		**Quality mean score:**		**RIH features mean score:**	
**Interpretation**<br>≥ 7/9 → Covers key aspects of RIH<br>< 7/9 → Compromised by missing information		**Interpretation**<br>≥ 2 → Based on superior quality sources<br>< 2 → Compromised by inferior quality sources		**Interpretation**<br>4.1-5.0: Almost all RIH features are present<br>3.1-4.0: Many RIH features are present<br>2.1-3.0: Few RIH features are present<br>1.0-2.0: Almost no RIH features are present	
**When one of the above two requirements is not met, the overall score is not meaningful**					

Figure 2. The scorecard

This Tool should be applied following the User Guide recommendations (infieri.umontreal.ca) and with proper academic citation. Silva, H.P., Lefebvre, A.-A., Oliveira, R.R., Lehoux, P. Fostering Responsible Innovation in Health: An evidence-informed assessment tool for innovation stakeholders. *Int J Health Policy Manag*. 2020; doi.10.34172/ijhmp.2020.34

© 2020 Silva, H.P., Lefebvre, A.-A., Oliveira, R.R., Lehoux, P.

Step 1: Screening

Inclusion Criteria	Potential information sources	Question to be answered in light of the available information
**Determinants of health**<br>Refer to the factors inside and outside the health system that determine health across one's life course. RIH should be **proven effective and safe** when addressing determinants of health, which include:<br>• Income and social status<br>• Social support networks<br>• Education and literacy<br>• Employment and working conditions<br>• Social or physical environments<br>• Personal health practises and coping skills<br>• Healthy child development<br>• Biology and genetic endowment<br>• Health services<br>• Gender<br>• Culture	• Type 1, Type 2 or Type 3 info indicating the effectiveness and safety of the innovation when it is used with the intended users	**Does the innovation effectively and safely address at least one determinant of health?**<br>• Yes, thus **include**<br>• No
**Innovativeness**<br>Refers to the degree of novelty of the innovation, which may entail solving a problem in a novel way, combining novel components, materials or social interventions, or new processes of production, distribution, commercialization or delivery. Considering the time it takes to develop a new sociotechnical solution, innovativeness should be appraised within the timeframe of the **past decade** and the context of use.	• Type 1 info describing the novelty of the innovation in view of the region where intended users are located	**Does the innovation address a problem in a novel way, is made of novel components, materials or social interventions, or is produced, distributed, commercialized or delivered in a novel way?**<br>• Yes, thus **include**<br>• No

Exclusion Criteria	Potential information sources	Question to be answered in light of the available information
**Unavailability**<br>At an early stage, an innovation may not be available in the form of a ready-to-use product, process or system. For instance, a prototype may have been developed to gather user feedback or a product may have been tested in clinical or community trials. When an innovation cannot be distributed or made available to its intended users, we recommend **postponing its assessment**.	• Type 1 info indicating that the innovation can be purchased or obtained in the region where its intended users are located	**Can the innovation be purchased or made available in the geographic region where its intended users are located?**<br>• Yes<br>• No, thus **exclude**
**Corporate Social Irresponsibility**<br>Refers to legal or illegal corporate actions that can harm people, animals or the environment. Examples of such actions may be observed in the following domains:<br>• **Animal welfare** (physical and psychological, wildlife habitats)<br>• **Community** (indigenous or local communities)<br>• **Diversity** (women or other underrepresented groups on board of directors or among senior managers)<br>• **Environment** (hazardous waste, toxic emissions)<br>• **Employees** (unions, workers' health and safety, retirement benefits)<br>• **Governance** (fiscality, managers' compensation, ownership, accountability)<br>• **Human rights** (labour rights, discrimination based on ethnicity, religion, gender or sexual orientation)<br>• **Products** (safety, marketing, antitrust)	• Type 2 info describing infringements in one domain of irresponsible corporate actions issued by governmental agencies, regulatory bodies or independent not-for-profit organizations that monitor human and labour rights, animal welfare and environmental regulation	**Has the organization that produces the innovation been involved in the past decade or is currently involved in irresponsible corporate actions?**<br>• No<br>• Yes, thus **exclude**

© 2020 Silva, H.P., Lefebvre, A.-A., Oliveira, R.R., Lehoux, P.

Step 2: Assessment

<table>
<tr><th rowspan="2">Assessment Attributes — Population health value</th><th rowspan="2">Potential information sources</th><th colspan="4">One option should be selected in light of the available information</th></tr>
<tr><th>A</th><th>B</th><th>C</th><th>D</th></tr>
<tr><td>Health relevance</td><td></td><td colspan="4">The innovation addresses a cause of death, injury or disability or a risk factor falling within…</td></tr>
<tr><td>Refers to the respective importance of the health needs addressed by the innovation within the overall burden of disease, considering the causes of death, injury and disability and associated risk factors in the region where the intended users are located.
Metrics of health relevance include number of deaths, disability-adjusted life years (DALYs), years lived with disabilities (YLDs), years of life lost (YLLs), prevalence and incidence rates.
Recent data for such measures (at a global, national or regional level) can be found in the Global Burden of Disease Study of the Institute of Health Metrics and Evaluation.</td><td>• Type 1 info describing the health needs addressed by the innovation
• Type 2 or Type 3 info analyzing the health needs addressed by the innovation</td><td>The top quarter of all causes of death, injury or disability or risk factors (75% and above)</td><td>The upper middle quarter (50% to 74%)</td><td>The lower middle quarter (26% to 49%)</td><td>The bottom quarter (the lowest 25%)</td></tr>
<tr><td>Ethical, legal and social issues (ELSIs)</td><td></td><td colspan="4">Means to mitigate the negative impacts of the innovation are available for…</td></tr>
<tr><td>Refers to an innovation's positive and negative impacts on the moral and sociocultural well-being of individuals and groups and to the legal and regulatory issues its use raises. Although not all ELSIs can be identified at an early stage, RIH considers the means by which negative impacts can be mitigated, which may include:
• For ethical issues: Patient decision-aids, psychological support, group empowerment, ethical guidelines, etc.
• For legal and regulatory issues: Laws and regulatory frameworks regarding individual rights, privacy, confidentiality, discrimination (health insurance, the workplace), adverse event monitoring, data stewardship, etc.
• For social issues: Stigma-reduction programs, caregiver support, community-led educational forums, return to work strategies, etc.</td><td>• Type 1 info describing the means to mitigate the negative impacts of the innovation
• Type 2 or Type 3 info analyzing how the negative impacts of the innovation can be properly mitigated in the region where the intended users are located</td><td>Nearly all applicable ELSIs</td><td>Several of the applicable ELSIs</td><td>Few of the applicable ELSIs</td><td>None of the applicable ELSIs</td></tr>
<tr><td>Health inequalities
Refers to the avoidable health status differences across individuals and groups that are associated with one's socioeconomic status, social position and capabilities (skills, knowledge, perceived self-efficacy, social network, etc.).
Groups who suffer a greater burden of mortality and morbidity due to who they are or where they grow up, live and work are considered vulnerable. Such groups include, but are not limited to:
• Subsistence farmers, long-term unemployed, informally employed, seasonal/daily workers
• People living in deprived urban or rural areas, living in poverty, experiencing homelessness, living with disabilities, living with mental illnesses
• Visible minority groups, asylum seekers, refugees, socially marginalized groups (e.g., lesbian, gay, bisexual, transgender and queer [LGBTQ+], low literacy, etc.)</td><td>• Type 1 info describing the intended users
• Type 2 or Type 3 info examining whether ability to benefit from the innovation varies across users due to one's socioeconomic status, social position or capabilities</td><td>The innovation…
Reduces inequalities by catering to the specific needs of a vulnerable group that are not met by current solutions</td><td>May contribute to the reduction of inequalities since ability to benefit from the innovation is not affected by one's socioeconomic status, social position or capabilities</td><td>May contribute to the increase of inequalities since the ability to benefit from the innovation is affected by one's socioeconomic status, social position or capabilities</td><td>Increases inequalities by catering to the specific needs of groups whose socioeconomic status, social position or capabilities are amongst the highest</td></tr>
</table>

© 2020 Silva, H.P., Lefebvre, A.-A., Oliveira, R.R., Lehoux, P.

Assessment Attributes — Health system value	Potential information sources	A (One option should be selected in light of the available information)	B	C	D
**Inclusiveness**		**Those who developed the innovation…**			
Refers to the degree of stakeholder engagement in the design, development and pilot stages of an innovation.<br>Different **methods** (e.g., codesign, interviews, citizen juries, focus groups, workshops, pilot testing, user assessment and feedback) can be used to engage different types of stakeholder (e.g., health and social care practitioners, decision makers, patients, relatives, community and civil society representatives).<br>Involving at an early stage a diverse and relevant set of stakeholders through an accountable method is likely to improve an innovation. Hence, RIH makes explicit the **rationale** and **scope** of the stakeholder engagement process and its **impact** on the innovation design and delivery.	• Type 1 info describing who was involved, why, how, when and with what impact<br>• Type 2 or Type 3 info analyzing who was involved, why, how, when and with what impact	Engaged a diverse and relevant set of stakeholders through a formal method and explained how their input was integrated in the design process	Engaged a diverse and relevant set of stakeholders through a formal method, but did not explain how their input was integrated in the design process	Either engaged a limited set of stakeholders or did not explain the method used	Did not engage stakeholders
**Responsiveness**		**The innovation addresses…**			
Refers to the ability to provide dynamic solutions to existing and emerging challenges in health systems.<br>To support health system sustainability, RIH should address **system-level challenges**, which may include:<br>• **Demographic shifts** (ageing, populations affected by climate change, war or conflicts)<br>• **Epidemiologic shifts** (chronic diseases, new or re-emerging infectious diseases, orphan diseases)<br>• **Human resources hurdles** (training, supervision, turnover)<br>• **Service delivery gaps** (accessibility, quality, patient centeredness)<br>• **Knowledge gaps** (data acquisition, analysis and interpretation, development and implementation of knowledge-based tools)<br>• **Governance gaps** (coordination, intersectoral action, community partnerships)	• Type 1 info describing the health system challenges being addressed and the region where the intended users are located<br>• Type 2 or Type 3 info analyzing the importance of the health system challenges in the region where the intended users are located	A system-level challenge that is documented as being of high importance in the target region	A system-level challenge that is documented as being of moderate importance in the target region	A system-level challenge that is documented as being of low importance in the target region	No specific system-level challenges
**Level and intensity of care**		**The innovation was designed for its use to take place mostly under the care of…**			
Refers to the principle of **subsidiarity** according to which the most decentralized unit in the health system, including the patient, should be mobilized to provide the service when it is possible to do so effectively and safely.<br>To support health system sustainability, RIH should seek to generate **high-quality outcomes while reducing labour intensity**. This may be achieved, for instance, by supporting patients' capacity for self-care, enabling proper follow-up by general practitioners, community health and social care providers, or reducing unnecessary interventions at the most specialized level of care of the health system.	• Type 1 info describing the level and intensity of care associated to the use of the innovation<br>• Type 2 or Type 3 info analyzing the level and intensity of care required for a safe and effective use of the innovation	The patient, an informal caregiver or a health and social care provider operating in a non-clinical environment	The patient, an informal caregiver or a health and social care provider operating in a primary health care facility	Health and social care providers operating in a secondary or intermediate level of care facility	Health and social care providers operating at the most specialized level of care within the health system

© 2020 Silva, H.P., Lefebvre, A.-A., Oliveira, R.R., Lehoux, P.

		One option should be selected in light of the available information			
**Assessment Attribute — Economic value**	**Potential information sources**	**A**	**B**	**C**	**D**
**Frugality**		**The innovation incorporates…**			
Refers to the ability to deliver greater value to more people by using fewer resources such as capital, materials, energy and labour time. Designers of frugal innovation aim to substantially reduce the costs of production and use of an innovation, focus on the core functionalities its users require and optimize its performance level considering the intended purpose and context of use.<br>Frugality may thus increase the economic value of RIH by incorporating three **characteristics**:<br>• **Affordability**, which may result from optimized innovation production processes and/or lower maintenance needs<br>• **Focus on core functionalities and ease of use** in order to meet the requirements of a larger number of users (e.g., in remote or resource-poor settings, at home, etc.)<br>• **Optimized performance**, which maximizes the fit between an innovation's characteristics and its context of use (e.g., robustness if used in difficult climatic conditions, transportability if used in remote settings, economies of scale if used in large centers, etc.)	• Type 1, Type 2 or Type 3 info describing the innovation's core functionalities, usability and costs, and the resources required for its production, utilization and maintenance	All three characteristics of frugal innovation	Two characteristics of frugal innovation	One characteristic of frugal innovation	No characteristics of frugal innovation

		One option should be selected in light of the available information			
**Assessment Attribute — Organizational value**	**Potential information sources**	**A**	**B**	**C**	**D**
**Business model**		**The business model of the organization that produces the innovation possesses…**			
Refers to the components through which an organization creates, delivers and captures social and economic value. A business model typically entails a tension between the redistribution of financial returns to shareholders and the provision of a high-quality innovation.<br>The business model of organizations that seek to provide more value to users, purchasers and society may possess the following **characteristics**:<br>• Pursue a social and/or environmental mission, operate on a not-for-profit basis or reinvest the majority of the revenues in their mission (e.g., social enterprises)<br>• Make the innovation freely usable or exploitable by others (i.e., open source, product licensing waivers, do-it-yourself)<br>• Adopt a pricing scheme based on ability to pay or a redistributive logic (e.g. customers who "buy one, give one")<br>• Employ people with particular needs (e.g., low literacy, disabilities)<br>• Comply with social responsibility programs (e.g., Certified B Corporation, SA8000 standard for decent work, ISO26000 for social responsibility)	• Type 1or Type 2 info describing the organization's structure, pricing scheme and compliance with social and environmental responsibility programs<br>• Type 3 info examining the economic, social and environmental dimensions of the organization's business model	Three of the characteristics described or more	Two of the characteristics described	One of the characteristics described	None of the characteristics described

© 2020 Silva, H.P., Lefebvre, A.-A., Oliveira, R.R., Lehoux, P.

<table>
<tr><th rowspan="2">Assessment Attribute — Environmental value</th><th rowspan="2">Potential information sources</th><th colspan="4">One option should be selected in light of the available information</th></tr>
<tr><th>A</th><th>B</th><th>C</th><th>D</th></tr>
<tr><td rowspan="2">Eco-responsibility

Refers to a product, process or method that reduces the negative environmental impacts of an innovation, including any equipment required by its use (e.g., batteries).

RIH can be supported by attending to eco-responsibility concerns at key stages in the lifecycle of an innovation, which include:
• Raw material sourcing (e.g., product or hardware made of recycled or renewable content materials, free of substances such as latex, metals or chemicals that are of major public health concern or harmful and toxic to ecosystems)*
• Manufacturing (e.g., efficient energy consumption, compliance with national or international environmental regulations, reduced solid or water waste)
• Distribution (e.g., packaging, transportation)
• Use (e.g., efficient energy consumption, reusability, durability)
• Disposal (e.g., product or hardware designed to be recycled, disassembled, remanufactured, composted or biologically degraded)
* Arsenic, asbestos, benzene, bisphenol A, bromine & chlorine-based compounds, cadmium, chromium, dioxin & dioxin-like substances, lead, mercury, phthalate, PVC.</td><td rowspan="2">• Type 1 info describing how environmental aspects are handled along the innovation's lifecycle
• Type 2 info describing how environmental certifications or requirements are fulfilled
• Type 3 info assessing the innovation's environmental impact along its lifecycle</td><td colspan="4">The innovation was designed by integrating eco-responsibility concerns at…</td></tr>
<tr><td>Three key lifecycle stages or more</td><td>Two key lifecycle stages</td><td>One key lifecycle stage</td><td>None of the key lifecycle stages</td></tr>
</table>

© 2020 Silva, H.P., Lefebvre, A.-A., Oliveira, R.R., Lehoux, P.

Step 3: Rating

Availability of information	Interpretation of the number of attributes with available information	
It is important to establish whether the information used to apply the Tool is sufficient. To this end, the **scorecard** indicates the number of attributes for which information was available.<br>We consider that the assessment relies on a sufficient number of attributes when at least 7 of the 9 attributes were documented.	**< 7/9: Insufficient number of attributes documented**<br>→ The assessment is compromised by missing information	**≥ 7/9: Sufficient number of attributes documented**<br>→ The assessment covers key aspects of RIH
**Quality of the sources of information**	**Interpretation of the quality of the sources of information**	
The scorecard indicates the sources of information used to score each attribute and the points associated to these sources.<br>If more than one type of information is used for an attribute, the **source of highest quality** is retained and rated as follows:<br>• Type 1. Low quality = 1 point<br>• Type 2. Moderate quality = 2 pts<br>• Type 3. High quality = 3 pts<br>The overall **quality of the sources of information** is determined by calculating the mean value of the points obtained, that is, the sum of the points obtained for each attribute (x_1, x_2, x_3, …, x_n) divided by the number of attributes with information available (n):<br>$\bar{x} = \frac{1}{n}\left(\sum_{i=1}^{n} x_i\right) = \frac{x_1 + x_2 + \cdots + x_n}{n}$	**< 2: Low to moderate quality**<br>→ The assessment is compromised by sources of information of inferior quality	**≥ 2: Moderate to high quality**<br>→ The assessment is based on sources of information of superior quality

Responsibility features of the innovation	Interpretation of the overall responsibility features score			
The attributes rely on a four-level Likert-like scale, where:<br>• A = a high degree of responsibility (5 pts)<br>• B = a moderate degree of responsibility (4 pts)<br>• C = a low degree of responsibility (2 pts)<br>• D = no particular signs of responsibility (1 point)	**4.1-5.0**<br>**Almost all RIH features are present**	**3.1-4.0**<br>**Many RIH features are present**	**2.1-3.0**<br>**Few RIH features are present**	**1.0-2.0**<br>**Almost no RIH features are present**
The **overall responsibility features score** of the innovation is determined by calculating the mean value of the points obtained, that is, the sum of the points obtained for each attribute (x_1, x_2, x_3, …, x_n) divided by the number of attributes with information available (n):<br>$\bar{x} = \frac{1}{n}\left(\sum_{i=1}^{n} x_i\right) = \frac{x_1 + x_2 + \cdots + x_n}{n}$	To interpret this score, one **must consider** whether the assessment relies on: a) a sufficient number of documented attributes (≥7/9); <u>and</u> b) information sources of superior quality (≥2).<br>**→ When one of these two requirements is not met, the score is not meaningful.**			
